# Babbling opens the sensory phase for imitative vocal learning

**DOI:** 10.1073/pnas.2312323121

**Published:** 2024-04-15

**Authors:** Albertine Leitão, Manfred Gahr

**Affiliations:** ^a^Department of Behavioural Neurobiology, Max Planck Institute for Biological Intelligence, 82319 Seewiesen, Germany

**Keywords:** song learning, birds, babbling, language acquisition

## Abstract

Zebra finches, a species of songbirds, learn to sing by creating an auditory template through the memorization of model songs (sensory learning phase) and subsequently translating these perceptual memories into motor skills (sensorimotor learning phase). It has been traditionally believed that babbling in juvenile birds initiates the sensorimotor phase while the sensory phase of song learning precedes the onset of babbling. However, our findings challenge this notion by demonstrating that testosterone-induced premature babbling actually triggers the onset of the sensory learning phase instead. We reveal that juvenile birds must engage in babbling and self-listening to acquire the tutor song as the template. Notably, the sensory learning of the template in songbirds requires motor vocal activity, reflecting the observation that prelinguistic babbling in humans plays a crucial role in auditory learning for language acquisition.

## Significance

Young birds learn their songs by listening to and imitating adult singers (the so-called tutor), often their father. This learning process involves a stepwise progression, where the birds initially memorize sounds they hear and later imitate the sounds by gradually refining their initial babblings to resemble the originally memorized tutor song. Babblings were thought to start the process of song imitation. In difference, we show now that babblings actually initiate the process of tutor song memorization. This finding parallels the notion that babbling facilitates language acquisition in humans.

**Fig. 1.**
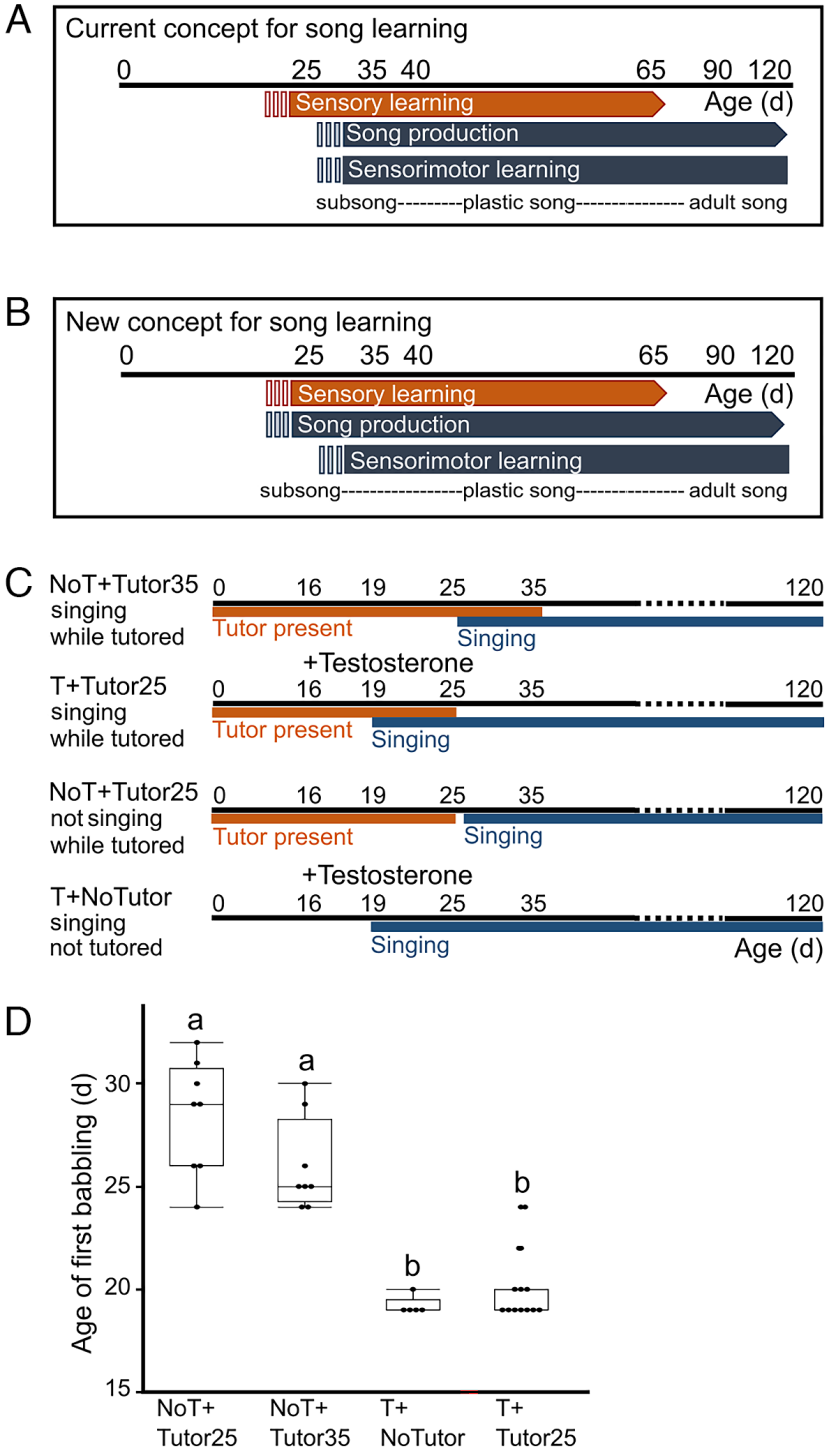
Testosterone treatment leads to an early onset of babbling, allowing experimental testing of a new conceptual framework for song learning in songbirds. In the conventional model (*A*), song sensory learning is believed to precede babbling production. However, our proposed model (*B*) suggests that babbling production plays a crucial role in initiating sensory learning of song. The experimental groups depicted in (*C*) include testosterone (T)-treated juvenile males exposed to a tutor until 25 d of age (d) (T + Tutor25), which corresponds to the natural onset of babbling; T-treated males without a tutor (T + NoTutor); non-T-treated males tutored until 25 d (NoT + Tutor25); and non-T-treated males tutored until 35 d (NoT + Tutor35), a known period sufficient for tutees to memorize their tutor’s song. Box-plots (*D*) showing that testosterone treatment at 16 d led to early babbling, starting as early as 19 d, regardless of tutor presence (T + Tutor25: mean ± SD, 20 ± 2 d, n = 12; T + NoTutor: 19 ± 0 d, n = 5). In contrast, non-T-treated males began babbling at later stage, with NoT + Tutor25 males starting at 28 ± 3 d (n = 8) and NoT + Tutor35 males starting at 26 ± 2 d (n = 8). These results demonstrate a significant difference in the timing of babbling between T-treated and non-T-treated males [ANOVA, F(3,29) = 39.93; *P* < 0.0001]. Experimental groups not connected by the same letter differ significantly. Each data point represents an individual bird.

Similar to human infants, young songbirds learn their songs by imitating vocalizations of adults (1–3). They begin, during a sensory learning phase, by forming a particular auditory memory of the sounds they hear from an adult tutor, often their father, which serves as the template for their own song learning. As they develop, this template guides them in refining their own vocalizations through sensorimotor feedback, gradually aligning their produced sounds with the template (sensorimotor learning phase). The early utterances of song precursors of songbird juveniles, known as “babblings” or “subsongs,” have traditionally been thought to initiate the sensorimotor phase of song learning, given the presence of a suitable model for memory formation (4). However, the mechanisms underlying the onset of the sensory phase of song learning remain elusive; the current view is that the onset of the sensory phase of vocal learning precedes babbling (1, 3, 5–7). In humans, even early forms of babbling during the first months after birth are important for some aspects of language development (8–11). This disparity makes motor-based learning models commonly applied to human language development, such as learning by synthesis (8, 9) or the articulatory filter hypothesis (9–11), inapplicable to songbirds. Although vocal–motor areas of songbirds do contribute to the encoding of auditory memories (12, 13), this does not challenge the observation that template learning precedes babbling. Thus, the formation of templates prior to the onset of babbling in birds would require specific auditory predispositions. The ontogenetic timing of template learning in juvenile birds represents a specialized differentiation process, distinct from general perceptual maturation of the auditory system. In zebra finches, the primary model for studying song-learning neural mechanisms (14), juveniles possess the ability to perceive complex sounds, including songs, prior to their own song learning (6, 15), and female zebra finches, despite not singing, can memorize their father’s song during development (7, 14). In this study, we re-evaluate the onset of template formation during ontogeny and test the hypothesis that the sensitive phase for song learning in zebra finches begins with, but does not precede, the bird's own babbling (Fig. 1*A*). Such a scenario would facilitate the development of mechanistic models to identify the neural equivalent of the template and would parallel the mechanisms expected for human language learning (8–11). To investigate this, we experimentally manipulate the ontogenetic onset of babbling in zebra finches using testosterone treatment, as testosterone influences song development in songbirds (16, 17). We then analyze the timing of sensory and sensorimotor learning in relation to the onset of babbling.

## Results

### Testosterone Treatment Resulted in an Early Onset of Babbling in Juvenile Males.

We manipulated the onset of babbling in male juveniles by administering either testosterone (T, n = 17) or empty implants (NoT, n = 16) at 16 d after hatching (d). The birds were raised with the mother and exposed to a tutor (father) until different time points: 25 d (Tutor25), which corresponds to the natural onset of singing, 35 d (Tutor35), which provides sufficient exposure for good imitation, or they were not exposed to any tutor (NoTutor). This led to four experimental groups: 1. “T + Tutor25” (n = 12); 2. “NoT + Tutor25” (n = 8); 3. “T + NoTutor” (n = 5); and 4. “NoT + Tutor35” (n = 8). Testosterone levels were only transiently elevated in the T + Tutor25 and T + NoTutor animals and returned to levels similar to the control animals (NoT + Tutor25 and NoT + Tutor35) within 2 wk or less after implantation (*Materials and Methods* and *SI Appendix*, Table S1). Given that first babblings are infrequent events with low amplitude and highly variable patterns (*SI Appendix*, Fig. S1), all-day recordings were essential to detect them accurately, as they can be easily missed by human observers. The literature reports a wide range of babbling onset from 25 to 40 d (17–19), which contrasts with our findings of a natural onset at 27 ± 3 d (NoT, mean ± SD, n = 16) in untreated males. Testosterone treatment at 16 d induced babbling as early as 19 d (this study and ref. 17), whether the juveniles were exposed to a tutor until day 25 (T + Tutor25: 20 ± 2 d; Fig. 1*C*) or not (T + NoTutor: 19 ± 0 d; Fig. 1*C*). In comparison, NoT + Tutor25 juveniles began to babble at 28 ± 3 d, and NoT + Tutor35 juveniles began at 26 ± 2 d. Thus, babbling occurred significantly earlier in testosterone-treated birds compared to non-testosterone-treated birds [ANOVA, F(3, 29) = 39.93; *P* < 0.0001]. The premature babblings induced by testosterone in T + Tutor25 did not significantly differ from the naturally occurring ones in terms of the analyzed sound parameters, including duration, pitch, goodness of pitch, and Wiener entropy (*SI Appendix*, Table S2).

### Testosterone-Dependent Early Onset of Babbling Triggers Sensory Learning of Songs.

**Fig. 2.**
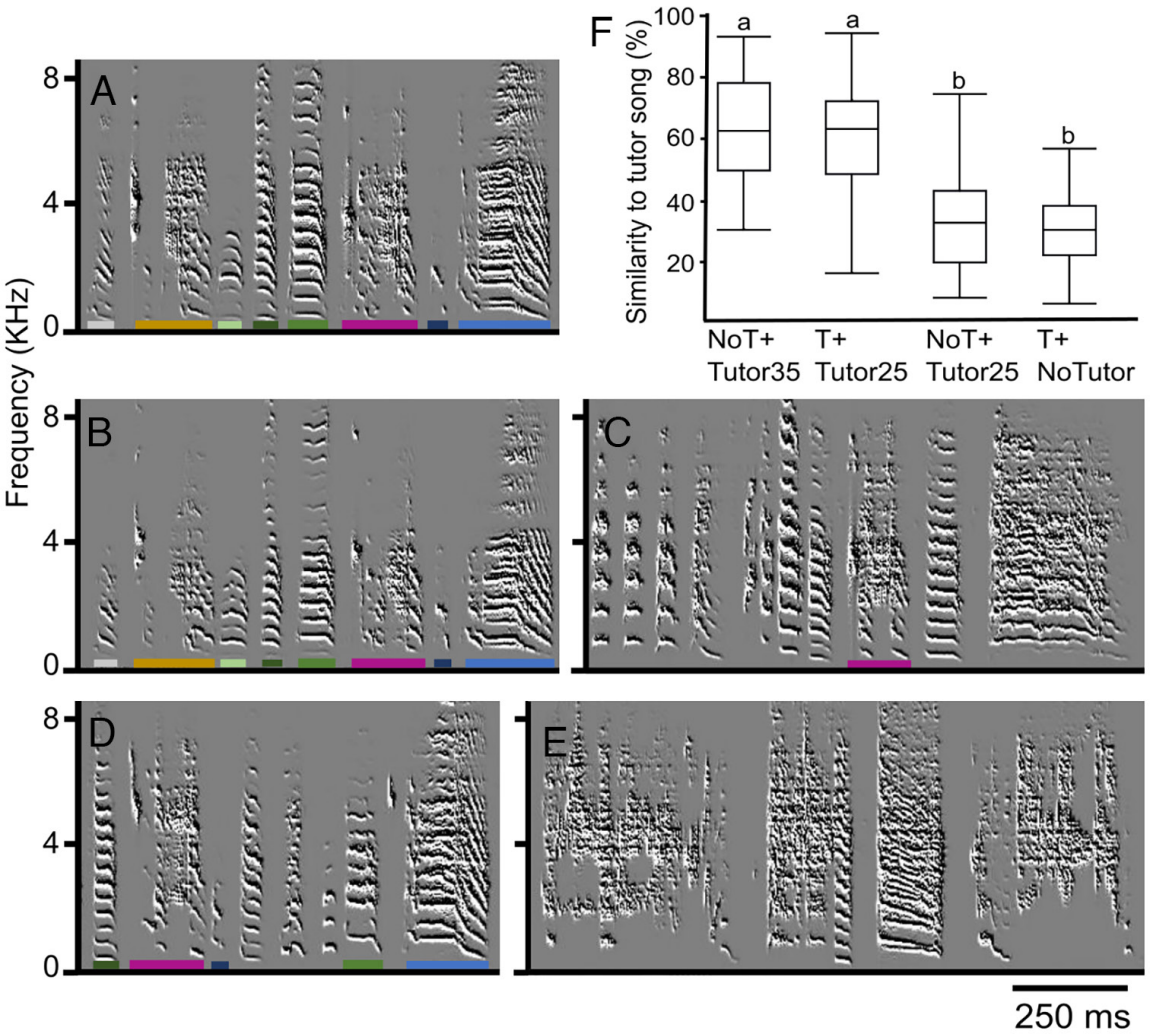
Babbling enables the learning of the tutor song. Sonagrams of song motifs are provided for a tutor (A) and its tutees as adults (*B*–*E*), with color-coded syllables indicating their similarity. The motif of the T + Tutor25 tutee (*B*) and the NoT + Tutor35 tutee (*D*) showed significantly higher similarity to the tutor motif (*A*) compared to the motifs of tutees who did not babble during tutoring (NoT + Tutor25, *C*) and males not exposed to a tutor (T + NoTutor, *E*). Even when exposed to the tutor song only until 25 d (the hypothetical natural onset of the song sensory learning period), testosterone-treated males who babble as juveniles form a memory of the tutor song (template). In (*F*), box plots show the mean percentage similarity between tutees and tutors song motifs for the experimental groups. The mean motif similarity of T + Tutor25 (%Similarity: mean ± SD, 61 ± 17%) is similar to that of NoT+Tutor35 (63.7 ± 16.6%) and significantly higher than those of NoT + Tutor25 (33 ± 15%) and T + NoTutor (31 ± 12%) [Full Factorial Repeated Measures F(3,29) = 10.7114, *P* < 0.0001, Tukey HSD post hoc test]. Values above 50% similarity indicate learning. Experimental groups not connected by the same letter differ significantly. For abbreviation of experimental groups, refer to the legend of Fig. 1.

We examined whether the early onset of babbling triggers sensory learning of songs. We quantified song learning by comparing the song motifs sung by the tutors and the tutees as adults (120 d) using %Similarity measurement of Sound Analysis Pro [SAP 2011, https://soundanalysispro.com/, (20)]. Previous work has shown that sensory learning of song in zebra finches occurs after 25 d (19). Juveniles in the T + Tutor25 and NoT + Tutor25 groups were exposed to the tutor until day 25, their putative sensory period (Fig. 1*C*), but T + Tutor25 juveniles produced babblings while NoT + Tutor25 juveniles did not. Importantly, T + Tutor25 birds learned the tutor’s song, whereas NoT + Tutor25 birds, who were exposed to the tutor’s song but did not sing themselves, did not learn it (Fig. 2).

T + Tutor25 juveniles imitated the tutor’s song as well as the control NoT + Tutor35 juveniles, who were exposed to the tutor between the onset of natural babbling and day 35 (Fig. 2), a period known to be sufficient for good imitation (19). The proportion of learned motifs (%Similarity: mean ± SD) in T + Tutor25 males (61 ± 17%) was similar to that of NoT+Tutor35 males (63.7 ± 16.6%) and fell within the range observed in zebra finches exposed to a tutor throughout ontogeny (21). In contrast, birds that did not sing during the tutoring phase showed poor imitation of the tutor’s song (NoT + Tutor25, 33,5 ± 15%, Fig. 2*F*), similar to birds that were not exposed to a tutor at all (T + NoTutor, 31 ± 12%, Fig. 2*F*) (repeated measures full factorial design for the four groups F(3, 29) = 10.7114, followed by LS-means differences Tukey HSD post hoc test, *P* < 0.0001).

Singing earlier due to testosterone treatment enabled template formation in T + Tutor25 birds at an earlier stage. Hearing one’s own babbling might play a role in gating the auditory pathway to allow neural responses to adult singing during this time window. However, the occurrence of one’s own babbling and tutor’s singing within a narrow time window does not appear to be relevant for song memorization, as there was no strong relationship between tutee’s babbling and the tutor’s singing events (*SI Appendix*, Fig. S3). Overall, these findings suggest that the early onset of babbling induced by testosterone enables template formation in zebra finches.

### Testosterone Treatment without Babbling Does Not Open the Sensory Learning Phase.

**Fig. 3.**
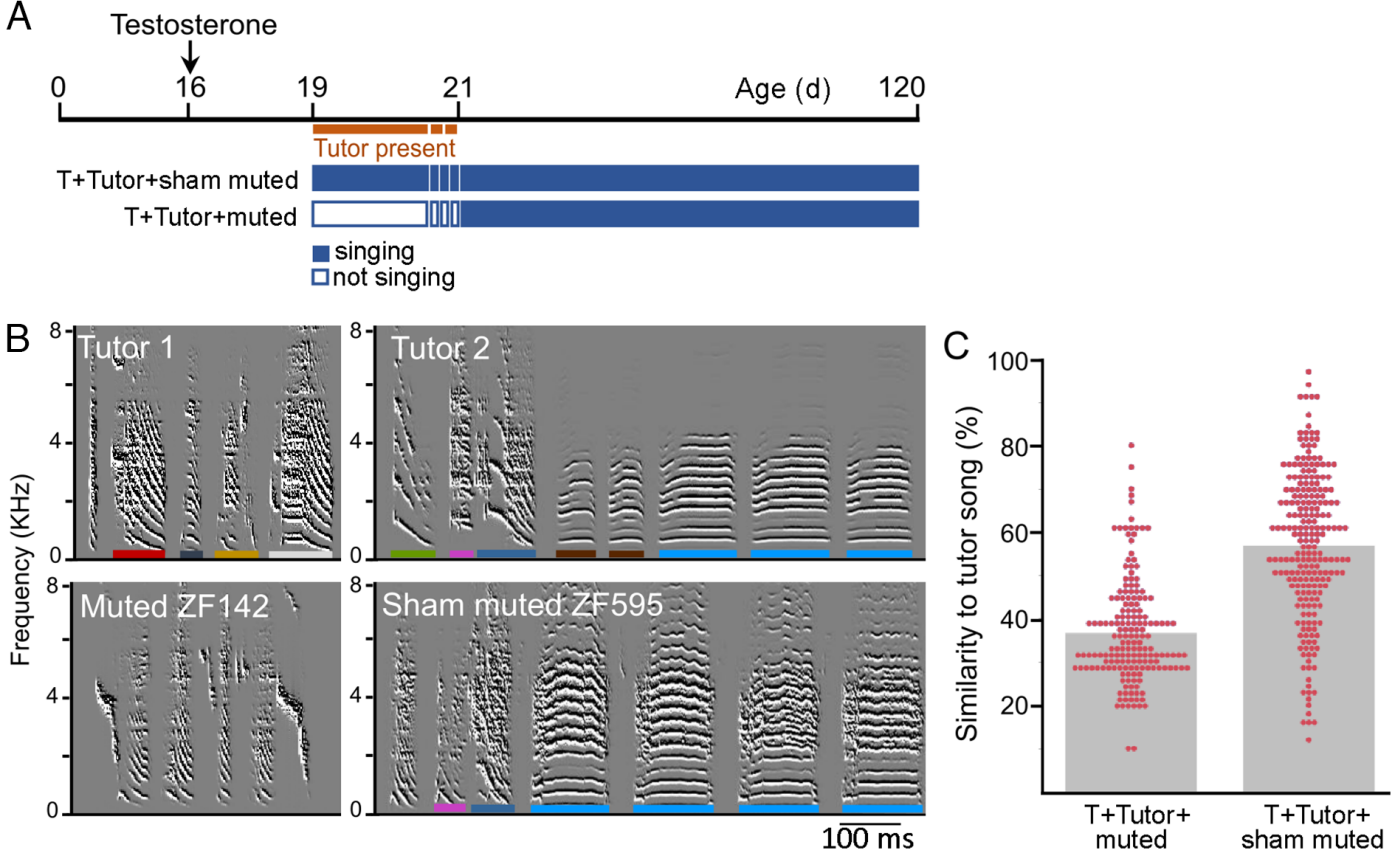
Learning of the tutor song is enabled by babbling, not by testosterone. (*A*) Timeline of the muting experiment. Juvenile males were treated with testosterone at 16 d of age (d). Babbling was interrupted in one cohort (T + Tutor+muted, n = 7) by puncturing the interclavicular air sac membrane, while it remained intact in the control group (T + Tutor+sham-muted, n = 9). The tutor was removed once the muted birds resumed their babblings after air sac membrane regeneration and tutor exposure was age-matched for the control birds. (*B*) Examples of sonograms of motifs of two tutors and their corresponding tutees whose songs were recorded as adults at 120 d. The muted ZF142 did not copy syllables from Tutor 1 while the sham-muted ZF595 copied syllables from Tutor 2. Tutor syllables are color-coded, and the tutee’s copied syllables are indicated using the same color code. (*C*) Motifs of the T + Tutor + muted tutees were significantly less similar to the tutors’ motifs (%Similarity: mean ± SD, 36.7 ± 12.1%) compared to the control tutees (T + Tutor + sham-muted: 56.8 ± 17%, Full Factorial Repeated Measures, F(1,14) = 10.0480, *P* = 0.0068, Tukey HSD post hoc test). Similarities above 50% are considered indicative of learning. The gray bars represent the means, each point represent individual %Similarity values.

We found that early babbling coincides with early formation of the template. Independent of its effect on the onset of babbling, testosterone might facilitate the formation of the template by acting on auditory neurons expressing the androgen receptor, a cellular mediator of testosterone action. Such neurons are present in the vocal control areas and in the caudal nidopallium (22, 23). To investigate this possibility, we treated a group of age-matched juvenile birds with testosterone at 16 d but interrupted their vocal production at the onset of babbling (19 d) in one cohort (T + Tutor+muted, n = 7) and left the babbling production intact in the control group (T + Tutor+sham-muted, n = 9). Both groups were exposed to a tutor during the same time window. To silence the birds, we punctured the interclavicular air sac (24), and once they started babbling again due to the regeneration of the air sac membrane (at around 21 d), we removed the tutor. We observed that the adult songs (120 d) of the temporarily mute birds showed no similarity to their tutor songs, in contrast to the control birds. The similarity scores (%Similarity: mean ± SD) of the muted birds (T + Tutor + muted: 36.7 ± 12.1%) were significantly lower than those of the control birds (T + Tutor + sham-muted: 56.8 ± 17%) indicating a lack of song learning in muted birds [Full Factorial Repeated Measures, Mixed Model, F (1, 14) = 10,0480, *P* = 0.0068] (Fig. 3). The muted birds exhibited normal behavior and did not show any signs of distress. They were capable of vocal beak gestures for silent begging and occasionally produced audible begging calls. This suggests that mute juveniles were able to produce syrinx movements during silent babbling and begging, which occasionally resulted in sounds when the airflow was strong. Previous findings support the occurrence of silent babbling, as by mechanically disturbed syrinx in juvenile zebra finches produced air sac pressure patterns resembling audible song, indicating the utilization of their song control system during silent song (25). From the muting experiment, we conclude that efference copies of silent babblings alone are not sufficient to initiate template learning. The ability of testosterone-treated juveniles to imitate a tutor’s song requires hearing one’s own song. Therefore, the role of testosterone in template learning is not possible through direct effects on auditory mechanisms but requires auditory feedback from self-produced vocalizations.

### Early Babbling Did Not Trigger Earlier Sensorimotor Learning.

We investigated whether early babbling promotes the sensorimotor phase of song learning. The conversion of the auditory template into motor song memory requires auditory–motor feedback and thus song-like vocalizations (3). Because the syllables of zebra finches’ babbling are highly variable but exhibit typical zebra finch characteristics such as frequency modulations, there is always some degree of similarity between juvenile babbles and tutor songs (*SI Appendix*, Fig. S3). To avoid such false-positive results and ensure accurate analysis, we compared syllables produced by juvenile tutees not only with the tutor’s syllables but also with syllables from pseudo-tutors, which were songs that the juveniles had never heard. In the T + Tutor25 group, the similarity between tutee and tutor syllables was higher than similarity between tutee and pseudo-tutor syllables in songs uttered at 50 d and older [ANOVA F(1, 9) = 104.74; *P* < 0.0001; post hoc Tukey HSD test] (*SI Appendix*, Fig. S2), so that sensorimotor learning occured in the expected ontogentic period (26). This finding suggests that early babbling did not advance sensorimotor learning of songs but rather prolonged the duration of the babbling phase. In contrast to our moderate and transient treatment, chronic treatment of young males with high testosterone levels disrupts vocal learning and shortens the sensorimotor phase of singing (27). Our results indicate that the production of variable sounds during babbling does not, by itself, lead to sensorimotor learning as previously hypothesized (28, 29). We assume that the functionality of the descending motor pathway is necessary for sensorimotor learning, which occurs through the formation of synapses between the vocal control areas HVC (proper name) to RA (robust nucleus of the arcopallium) between 25 and 35 d (30).

## Discussion

We demonstrated that active babbling is necessary to initiate the critical sensory phase of song learning, that is the formation of the template, which subsequently guides song development through sensorimotor feedback mechanisms in young zebra finches. This babbling-dependent initiation of template learning is likely a widespread mechanism among songbirds, as many species begin babbling around the onset of their sensory phase of song learning (31, 32) (Table 1). The generation of babbling through intrinsic feed-forward motor models and the auditory reafferent input of self-generated babbling sounds likely induce the development of motor auditory maps in the zebra finches. These maps then enable the memorization of heard adult songs, similar to what has been proposed for language learning in human infants (8–11).

**Table 1.** The onset of babbling starts the phase of song sensory learning but does not advance song sensorimotor learning

Condition tested	Expectation	Result
Onset of testosterone induced vs. natural babbling. T vs. NoT	Wide range of onset of babbling is reported in the literature (25 d to 40 d).	Natural onset of babbling at ca. 27 d in untreated males as compared to 19 d in testosterone-treated males (Fig. 1).
Babbling onset and song learning. T + Tutor25 vs. NoT + Tutor25 and NoT + Tutor35	No relationship between tutee’s babbling and song learning.	Babbling onset triggers sensory learning of songs (Fig. 2).
Testosterone treatment without babbling. T + Tutor + muted vs. T + Tutor + sham muted	Testosterone alters the auditory system to facilitate sensory song learning.	Muted birds (no audible babbling) did not learn from tutors while sham muted controls (babbling and self-hearing) did (Fig. 3).
Early babbling and sensorimotor song learning. T + Tutor25 vs. NoT + Tutor35	Babbling promotes sensorimotor learning.	Early babbling did not advance sensorimotor learning but prolonged the babbling phase. Sensorimotor learning after 50 d, as expected from literature (*SI Appendix*, Fig. S2).

Testosterone = T. Experimental groups: T + Tutor25: T treated and exposure to a tutor until 25 d. NoT + Tutor25: Not T treated and exposure to a tutor until 25 d. T + NoTutor: T treated and no exposure to a tutor. NoT + Tutor35: Not T treated and exposure to a tutor until 35 d.

Previous studies of song development in zebra finches have indicated the involvement of the anterior forebrain circuit of the song-controlling neural network, in babbling (28, 33). However, the neuron pools in these areas have limited auditory properties (34), making them unlikely sites for template formation. Alternatively, it is possible that efference copies of motor activity associated with babbling are sent to the auditory cortex-like forebrain areas that contain auditory units responsive to complex song features (14). However, the premotor neuron pools of the anterior forebrain babbling pathway do not have strong projections to auditory forebrain areas (35). Another possibility is that the template is directly formed in the HVC, which is essential for sensorimotor learning and control of the adult song pattern (12). The HVC of adult zebra finches has synaptic connections with associated cortex-like auditory areas (36, 37) and dopaminergic mechanisms in the HVC facilitate template learning in certain context (38). However, the HVC does not appear to be involved in babbling (28), and the anterior forebrain circuitry does not project to the HVC (39). Therefore, complex multisynaptic pathways would be required to forward efferent information about babbling production and auditory information about one’s own babblings into the HVC for template formation to occur. Thus, the circuits that enable babble-induced template formation have yet to be uncovered. The overlapping nature of the sensory and sensorimotor learning phases in naturally singing zebra finches has made it challenging to distinguish between the template and memories resulting from sensorimotor learning, as they should be similar to each other. Since testosterone promoted template learning but not sensorimotor learning, the experimental model presented enables the disentanglement of these two mechanisms. Akin to previous androgen treatments of young male zebra finches that did not significantly alter the overall development of the song control system (40, 41), our transient testosterone treatment is unlikely to have affected the overall development of vocal-controlling brain regions. Similarly, ontogenetically late testosterone treatment is unlikely to affect the overall development of the vocal system, as suggested by hormone treatments in comparably aged female zebra finches (42) but suggests changes in certain cell types of the vocal control areas (43). Therefore, cell-based molecular biology methods are needed to identify the types of neurons that are affected by testosterone or its androgenic and estrogenic metabolites during the transient testosterone surge, thereby enabling babbling and subsequent sensory-motor integration for song sensory learning.

Parallels between birdsong learning and human speech learning have been commented on for a long time (1). In infants who do not babble or start babbling late due to anatomical or neurological reasons often exhibit difficulties in forming perceptual memories during their silent period, leading to limited vocal expressive vocabulary and delayed language skills (44–47). These observations, along with experimental findings, such as those caused by impediments to infant vocal articulation, and recognition that infants’ speech representations are sensorimotor in nature, as well as infants’ covert motor behavior, collectively suggest that the current perspective positing sensory learning occurs first in the absence of vocal–motor behavior needs to be revised to the view that infants’ speech representations are sensorimotor [for rewiew (9)]. The exact role of infants’ early vocalizations for speech learning, however, remains to be uncovered. The intricate stages of infant babbling should not be compared one-to-one with the babblings of songbirds. However, conceptual analogies exist concerning the integration of sensory and motor information enabling learning of vocal templates during early vocal development: The experience of zebra finches aged 19 to 25 d with their own vocal tract, which produces highly variable sounds containing elements that resemble parts or whole syllables of the tutor (*SI Appendix*, Fig. S2), suggests learning by synthesis or according to the articulatory filter hypothesis in songbirds, as has been proposed for human infants (8–11).

## Materials and Methods

### Animals.

Animal experiments were conducted following approved protocols by the government of Upper Bavaria (Az.55.2.1.54-2531-141-09 and ROB-55.2-2532 Vet. 02-18-155). The study included a total of 49 male zebra finches (*Taeniopygia guttata*) from which the results were collected. They were housed in cages (120 × 42 × 42 cm) in sound-proof chambers.

Experimental birds consisted of juvenile male zebra finches (tutees) raised in a natural parental care setting. At 16 d after hatching (d), the tutees were implanted with either a testosterone (T, n = 17) or an empty (NoT, n = 16) pellet (see below). The tutees were randomly assigned to four experimental groups: 1. “T + Tutor25” (n = 12): Tutees received testosterone treatment and were exposed to their tutor until 25 d. 2. “NoT + Tutor25” (n = 8): Tutees received an empty implant and were exposed to their tutor until 25 d. 3. “T + NoTutor” (n = 5): Tutees received testosterone and were without tutor but a female adult that does not sing. 4. “NoT + Tutor35” (n = 8): Tutees received empty pellets and were exposed to their tutor until 35 d. In addition, two experimental groups were introduced to investigate the effects of babbling prevention in testosterone-treated juveniles during exposure to their tutor: 1. T + Tutor + sham-muted (n = 9): Tutees were sham muted and were able to produce audible babbles, and 2. T + Tutor + muted (n = 7): Tutees were muted and not able to produce audible babbles. The muting procedure (24) was performed by disrupting the intraclavicular air sac under anesthesia (1% isoflurane), while the sham-muted tutees underwent a similar surgical treatment without disrupting the air sac. This technique prevented the birds from babbling for 2 to 5 d until the self-regeneration of the air sac membrane. The tutors were removed as soon as they resumed their first babblings and tutors were present during the same time window in the age-matched controls.

### Testosterone Implantation.

The pellets (Innovative research of America Sarasota, FL, USA) were either designed to contain 1.5 mg of testosterone that was released over a period of 21 d (testosterone group) or were empty placebos (non-testosterone group). For the implantation, we prepared the dorsal, lower-neck skin area by cleaning it with alcohol 70% and applied a topical anesthetic (lidocaine), a small incision (1 to 2 mm) was made with scissors. The pellet was then inserted subcutaneously, and the incision was sealed using veterinary glue.

### Testosterone Analysis.

Blood samples were collected from the wing vein at post-hatching days 20, 30, 40, 50, 60, 70, 80, and 90 of individuals within the four experimental groups (*SI Appendix*, Table S1), using heparin-coated microhematocrit capillaries. The plasma was separated by blood centrifugation at 2,500 rpm for 10 min and stored at −80° C until subsequent analysis. For the quantification of testosterone levels, a custom-made radioimmunoassay (RIA) was used following the procedures previously published (48). In brief, testosterone was extracted from plasma with a recovery rate of 0.91% ± 0.04% (mean ± SD). The lower limit of detection of the standard curves was determined as the first value outside the 95% CI for the zero standard (Bmax), resulting in a limit of detection for testosterone of 6.5, 8.9, and 7.3 pg/mL per RIA, respectively. The interassay coefficient of variation of the three RIAs was 4.2. All samples fell within the detectable ranges for testosterone. Testosterone concentrations differed significantly between the T-treated groups (T + Tutor25, T + NoTutor) and the control groups (NoT + Tutor25, NoT + Tutor35) only 20 d after hatching [mixed model, F(21, 173) = 8.0345, *P* < 0.0001; see *SI Appendix*, Table S1 for details].

Statistical analysis of testosterone concentrations was conducted in R and JMP Pro. We used a Full Factorial Design with a mixed model to investigate variations in testosterone concentrations (pg/mL) across distinct experimental groups at specific post-hatching ages to simultaneously consider the influence of multiple factors, providing understanding of the interactions between experimental groups and posthatching sampling dates. Subsequently, post hoc analysis using Tukey’s HSD test was conducted to discern specific pairwise differences between the experimental groups.

### Sound Recording and Analysis.

Vocalizations were recorded automatically using Sound Analysis Pro software [SAP 2011, https://soundanalysispro.com/ (20)] from 16 d until adulthood (120 d). The default settings of SAP were applied during the processing of the vocalizations, which were sampled at a rate of 44.054 KHz. The recording setup consisted on a PC equipped with an Edirol UA1000 sound card (16-bit, 44.1 kHz) connected to an Earthworks TC20 multidirectional microphone positioned above the cage.

For the comparison of syllable parameters on the first day of babbling across the different experimental groups, we first, manually delineated all the babbles produced on the first day of babbling to ensure that they did not overlap with the vocalizations of the parents. Subsequently, we used SAP to automatically segment the syllables by adjusting the thresholds for amplitude and Wiener entropy. This automated segmentation allowed us to measure the duration of each syllable as well as four spectral features: pitch, goodness of Pitch, frequency modulation (FM), and Wiener entropy.

To measure the similarity between the song motifs of tutees and tutors, we conducted 25 comparisons using representative song motifs (excluding introductory notes) of juveniles recorded at 120 d. The calculation of the percentage similarity (%Similarity) was performed using the SAP software. For the T + NoTutor group, the %Similarity was calculated by comparing the song motifs of tutees with those of randomly selected adult males (referred to as pseudo-tutors).

We compared the syllables of tutees and their respective tutors at different time points through development: first-day babbling, 25 d, 30 d, 50 d, and 120 d. This analysis specifically focused on tutee–tutor pairs from the T + Tutor25 group, with a particular emphasis on tutees who exhibited motif %Similarity scores above 50%. To identify the target syllable, we initially examined the tutee’s motifs at 120 d and identified a specific syllable that had %Similarity to the tutor syllable higher than 50%. We then traced back through the tutee’s song development to find the corresponding syllable in the 50 d motifs that was similar to the 120 d target syllable. This process was repeated for the 30 d, 25 d, and first-day babbling time points. In cases where we could not find a matching syllable in the tutee’s motif, we randomly selected syllables from the tutee’s song recorded at that age. For each syllable type and age level examined, we selected 10 renditions and compared them to 10 tutor syllables, resulting in 100 tutee-tutor syllable comparisons for a given age and syllable type. To standardize the measurement of syllable similarity, we also compared tutee syllables with syllables from an unknown tutor (referred to as a pseudo-tutor). We quantified the %Similarity using SAP software.

We measured the duration of the silent intervals between the end of tutor’s song and the beginning of the tutee’s babbling and between the end of the tutee’s babbling and the beginning of the tutor’s song, for all tutees in the T + Tutor25 group. In cases where there were overlaps between tutee and tutor vocalization, we assigned a duration of 0 ms to the gap. These measurements of silent interval durations were performed using SAP software and included babblings uttered from the first day until 25 d.

Statistical analysis was conducted in R and JMP Pro. ANOVA and post hoc Tukey HSD tests were performed to evaluate main effects and interactions between experimental conditions and age in relation to the measured variables. We used a Full Factorial Repeated measures (ANOVA, mixed model) to investigate differences in similarity scores used to assess the song learning among the experimental groups. The repeated measures accounted for multiple observations from the same bird. Linear mixed-effects models were used for analyzing song development data, with the different developmental stages as dependent variables and the experimental groups as a fixed factor. We used generalized linear mixed-effects models (packages: glmmTMB for mixed model; pairwise comparisons) to investigate differences in syllable parameters (duration, FM, entropy, pitch, pitch goodness) of babblings recorded at the first day of babbling between the four experimental groups. Age (in days) of first babbling and Bird ID were included as random effects. *P* values were adjusted using the Tukey method for comparing a family of four estimates.

## Supplementary Material

Appendix 01 (PDF)

## Data Availability

Sound files data have been deposited in Albertine Leitão and Manfred Gahr, 2023; Dataset for the manuscript entitled “Babbling opens the sensory phase for imitative vocal learning” (https://doi.org/10.17617/3.3Y67P3). https://edmond.mpdl.mpg.de/privateurl.xhtml?token=716dd39b-1f13-4742-8841-52e09a31c83b (49).
