# Supplementary material for: Babbling opens the sensory phase for imitative vocal learning
Source: Proc Natl Acad Sci U S A. 2024 Apr 15;121(18):e2312323121. doi: 10.1073/pnas.2312323121 (PMC11067029; doi:10.1073/pnas.2312323121)
Supplement: Supplementary file 1 — Appendix 01 (PDF) [file pnas.2312323121.sapp.pdf]

SI Appendix

Babbling opens the sensory phase for imitative vocal learning.

Albertine Leitão and Manfred Gahr*

Department of Behavioural Neurobiology, Max Planck Institute for Biological Intelligence, 82319 Seewiesen, Germany.

Tab. S1

Tab. S2

Fig. S1

Fig. S2

Fig. S3

Tab. S1. Testosterone (T) plasma levels of the testosterone treated groups (T+Tutor25, T+NoTutor) differed from placebo treated males (NoT+Tutor25, NoT+Tutor35) only at post-hatching day (d) 20, i.e. for less than two weeks following T implantation (Post-hoc Tukey HSD following a Mixed Model analysis, $F(21, 173) = 8.0345$, $p < 0.0001$). The results underscore the temporal specificity of T dynamics within the examined experimental cohorts, excluding a long-term effect of T treatment on the ontogeny of T plasma profiles. T depots were implanted at 16 d. Blood plasma was sampled every 10 days starting at 20 d. Means ± standard deviations of T concentration levels are presented for each experimental group at specific post-hatching days (Day). The measurements were conducted on “N” individuals within the respective experimental groups: T+Tutor25 (N = 12): T treated and exposure to a tutor until 25 d. NoT+Tutor25 (N = 8): Not T treated and exposure to a tutor until 25 d. T+NoTutor (N = 5): T treated and no exposure to a tutor. NoT+Tutor35 (N = 8): Not T treated and exposure to a tutor until 35 d. In some groups, Ns are variable since we did not collect blood samples of all individuals.

Day	N	T+Tutor25	N	NoT+Tutor25	N	T+NoTutor	N	NoT+Tutor35
**20**	10	3441.2 ± 1664.1	8	61.1 ± 35.2	5	5475.5 ± 3557.3	8	88.7 ± 95.0
**30**	9	250.4 ± 143.4	8	92.0 ± 53.8	4	148.2 ± 66.8	8	81.9 ± 38.3
**40**	10	257.3 ± 308.0	8	157.4 ± 59.6	4	260.9 ± 239.0	8	94.8 ± 66.3
**50**	10	326.9 ± 374.4	8	483.5 ± 475.0	3	414.6 ± 233.9	8	554.2 ± 357.3
**60**	9	552.8 ± 729.0	8	779.1 ± 348.9	3	293.4 ± 221.8	8	440.7 ± 376.7
**70**	9	572.7 ± 620.8	8	570.0 ± 558.0	3	577.9 ± 176.8	8	886.5 ± 659.8
**80**	9	944.6 ± 813.9	8	786.0 ± 799.5	3	547.6 ± 421.0	8	1058.1 ± 652.8
**90**	8	1402.5 ± 1399.8	7	954.0 ± 826.9	3	939.3 ± 444.0	8	1522.4 ± 1330.4

Tab. S2. Sound variables of babbling syllables in different experimental groups. Means and standard deviations (sd) of various sound variables of babbling syllables in different experimental groups. These parameters were measured on 'N' babble syllables produced on the first day of babbling by juveniles of the different experimental groups: T+Tutor25: testosterone (T) treated and exposed to a tutor until 25 days. NoT+Tutor25: Not T treated and exposed to a tutor until 25 days. T+NoTutor: T treated and not exposed to a tutor. NoT+Tutor35: Not testosterone treated and exposed to a tutor until 35 days. The sound parameters include syllable duration (msec), pitch (Hz), frequency modulation (FM) (Hz), Wiener entropy, and goodness of pitch. Notably, the premature babblings induced by testosterone in T+Tutor25 did not differ significantly from the normally occurring babblings for the measured sound parameters, including duration, pitch, goodness of pitch, Wiener entropy and FM (generalized linear mixed-effects models (packages: glmmTMB for mixed model; pairwise comparisons)).

**Experimental group**		**Duration**		**Pitch**		**FM**		**Wiener Entropy**		**Goodness of pitch**	
	**N**	**Mean**	**sd**	**Mean**	**sd**	**Mean**	**sd**	**Mean**	**sd**	**Mean**	**sd**
NoT+Tutor35	2491	133.8	105.1	861.4	265.4	39.9	7.7	-2.4	0.5	229.8	66.8
NoT+Tutor25	875	105.9	84.8	791.1	279.7	39.0	9.4	-2.4	0.6	219.7	73.6
T+NoTutor	3627	173.9	130.2	747.8	205.0	37.7	6.6	-1.9	0.6	194.4	50.5
T+Tutor25	1368	148.9	115.0	814.5	233.5	36.8	8.6	-2.3	0.5	201.6	55.2

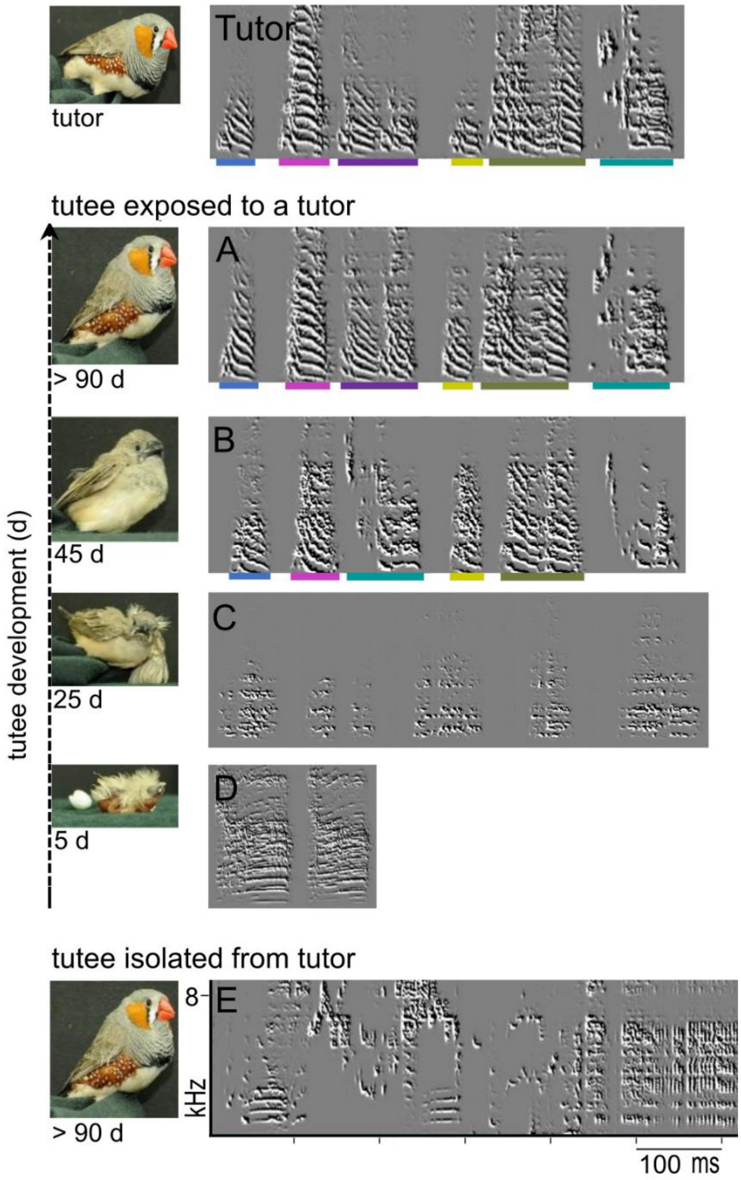

Fig. S1. Sonagrams showing the song ontogeny of male zebra finches. The top sonagram shows the motif of the tutor with color-coded syllables. The process of song learning is a gradual one (19, 27, 50). At early age (**D**) young males (tutees) produce innate begging calls around 3-5 days (d) to stimulate parental care. Around 25-30 d (**C**), they start producing highly variable, soft and rambling vocalizations, known as babblings, which lack resemblance to the tutor's syllables. As development progresses (**B**), around 45 d, recognizable yet variable syllables start to emerge, showing some similarities to tutor's syllables. The tutee's syllable variability gradually decreases as the song continues to develop (**A**), eventually matching the syllables of their tutor in adulthood (90 d or older). Color bars indicate the degree of resemblance between the tutee's syllables and those of the tutor. In **E**, we show the song of a juvenile raised in isolation from a tutor, exhibiting aberrant syllables. Abbreviations: ms: millisecond; kHz: kilohertz.

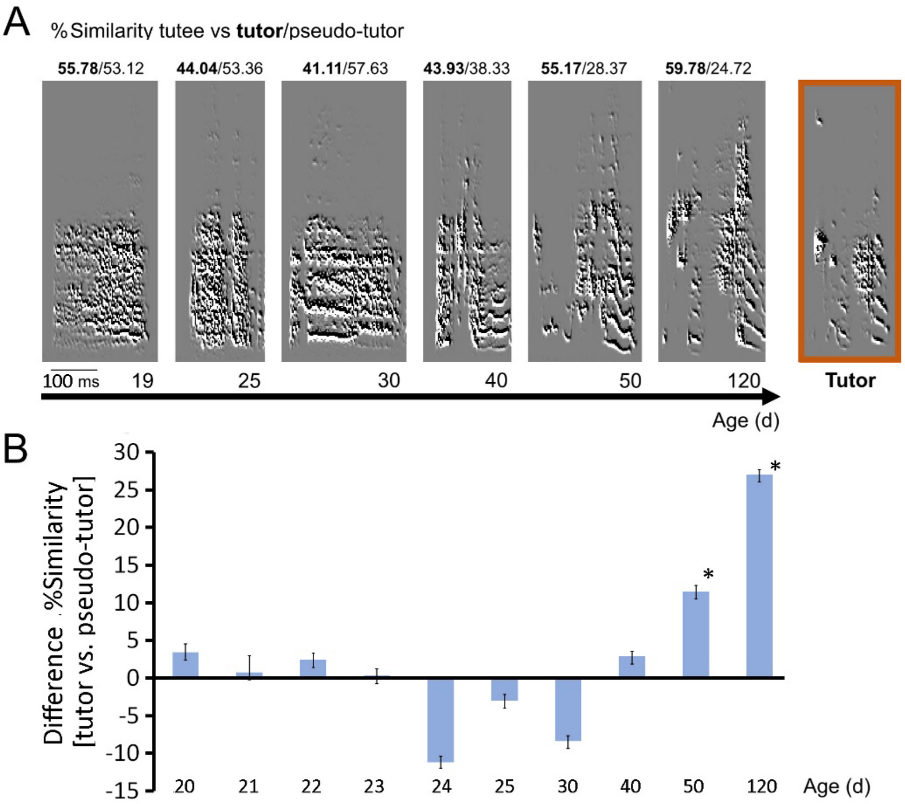

Fig. S2. Ontogeny of babble syllables and their similarity to syllables emitted by their tutor, as compared to a randomly pseudo-tutor that the tutee never heard. (**A**) The sonagrams depict representative examples of a tutee's syllables emitted between 19 days (d) and adulthood (120 d), compared to the target tutor syllable (orange box). The top of each sonagram displays the similarity (%Similarity) of the tutee's syllable to the tutor syllable (labeled in bold) and to the syllables of a pseudo-tutor. From 50 d onward, the syllables showed learned features with %Similarity to the target syllable above 50%. Note that the tutee syllable uttered at 19 d to 33 d shows a similarity above 50% with the pseudo-tutor, supporting the notion that some spontaneously uttered syllables have general similarities with syllables of adult zebra finches. In (**B**), the graphs illustrate the specific learning of syllables from tutors as compared to pseudo-tutors occurring from 50 d onward. The syllables of tutees (T+Tutor25 group) were compared to the syllables of their respective tutor and to the syllables of a pseudo-tutor. The resulting %Similarity for the tutee-tutor and the tutee-pseudo-tutor comparisons were subtracted. Shown are these mean differences (mean ± sd) for babblings recorded between 20 d and 120 d. Higher similarity to the tutor, as compared to the pseudo-tutor, emerged at 50 d and 120 d (ANOVA $F(1,9) = 104.74$, $p < 0.0001$; post hoc Tukey HSD test). * indicates statistical significance.

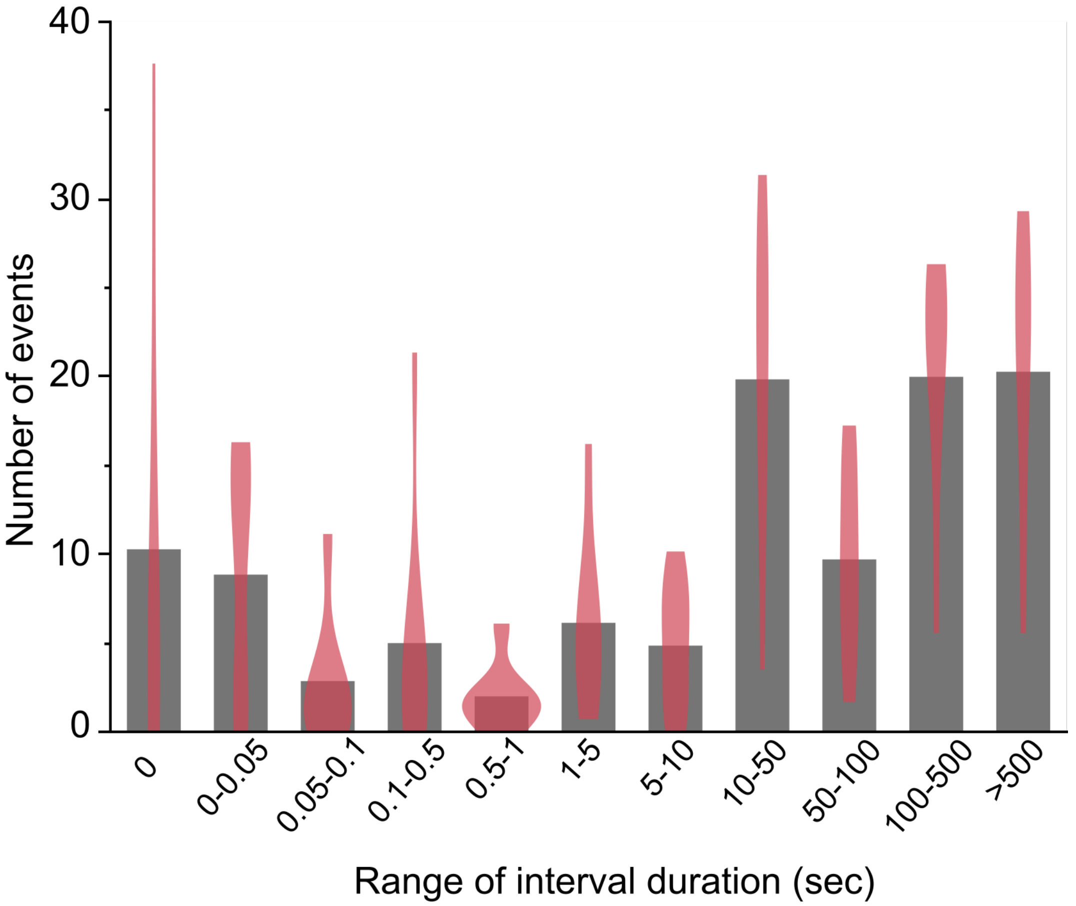

Fig. S3. Tutee and tutor rarely sing in a narrow time slot. Distribution of interval durations (in seconds, sec) between the songs of tutors and the babblings of T+Tutor25 tutees. This analysis includes babblings uttered from the first day of babbling until 25 days. The grey bars represent the mean number of events at different interval durations, while the red violin plots represent the summary statistics and density of each variable. The data reveals that tutees and tutors rarely sing in a narrow time slot, suggesting that the timing of their vocalizations does not appear to be significant for template learning. Fifteen percent of the intervals were less than 0.5 sec, approximately, 21% between fell between 0.5 sec and 10 sec. The majority, 64% of the intervals exceeded 10 sec. An interval of 0 sec corresponds to overlaps between tutor song and tutee babble.
